# Supplementary material for: Windows of developmental sensitivity to social media
Source: Nat Commun. 2022 Mar 28;13:1649. doi: 10.1038/s41467-022-29296-3 (PMC8960761; doi:10.1038/s41467-022-29296-3)
Supplement: Supplementary file 1 — Supplementary Information [file 41467_2022_29296_MOESM1_ESM.pdf]

Supplementary Information for “Windows of developmental sensitivity to social media”

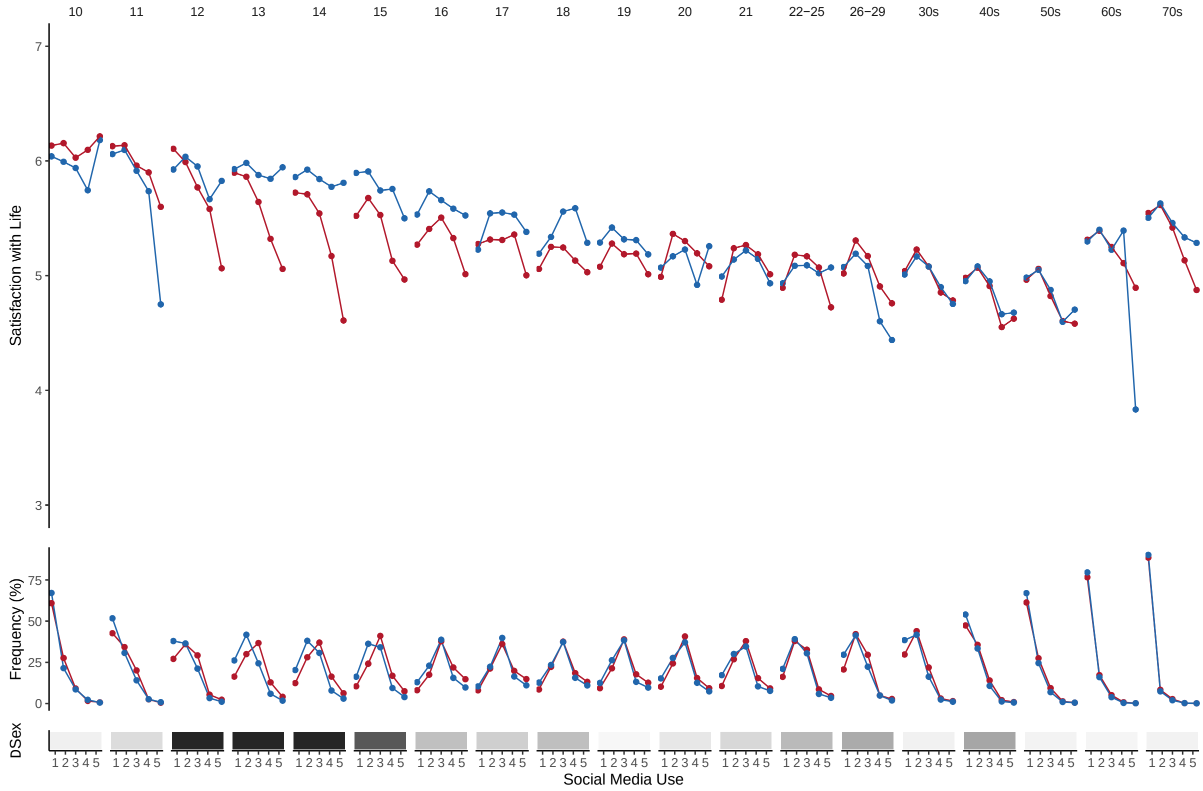

Supplementary Figure 1: Estimated social media use and life satisfaction ratings across the lifespan (Extended version)

Top: Cross-sectional correlation between estimated social media use and a one-item satisfaction with life measure for 72,287 UK participants of the Understanding Society dataset between the age of 10 and 80 years. The results are split by age and sex: female = red, male = blue. The 95% confidence intervals represent the lower and upper Gaussian confidence limits around the mean based on the t-distribution. Middle: Frequency distribution of estimated social media use by age and sex. Bottom: Shading of each rectangle represents whether a model relating estimated social media use and life satisfaction ratings that takes into account a possible sex difference is more likely to represent the data than a model that does not take into account sex: darker shade = model with sex differences is more likely.

It should be noted that as high levels of social media use are very rare in the youngest and oldest age groups present in the data (e.g., ages 10, 11 and 60+, Supplementary Figure 1), we cannot evaluate functional form in these groups. Further, as most participants were measured multiple times, more than one data point per participant will appear in this graph. Source data for this figure are provided as a Source Data file.

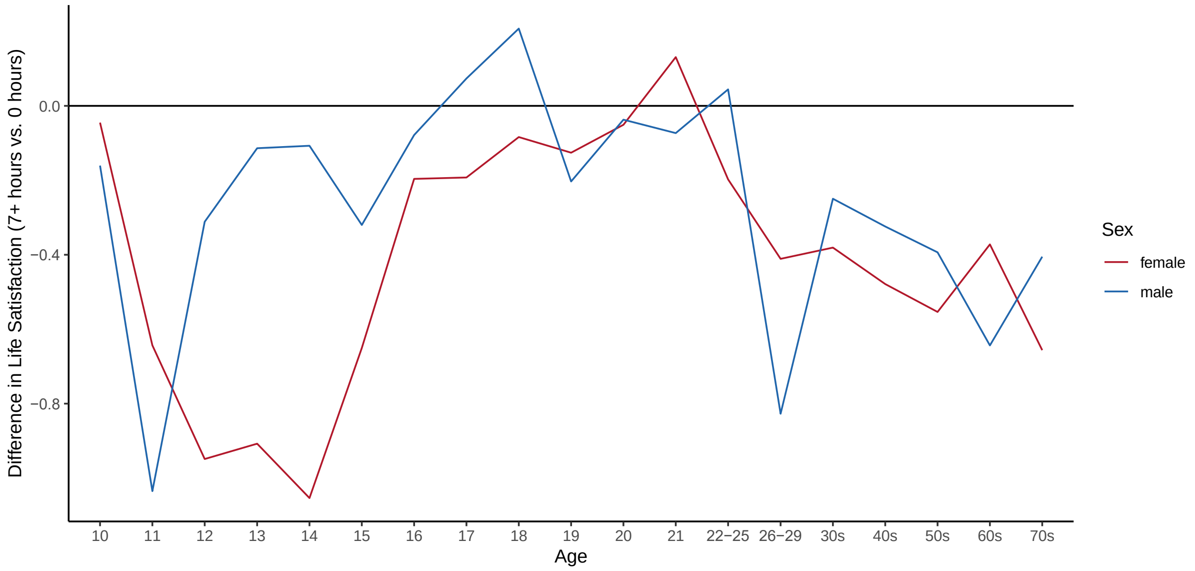

Supplementary Figure 2: Difference between the predicted life satisfaction of those participants who report using 7 or more hours of social media vs those who report using no social media

The graph plots the difference between the model predicted life satisfaction ratings for those who report using 7+ hours of social media (score: 5) and those using no social media (score: 1) for different age groups (shown on the x axis). The model was a linear regression predicting life satisfaction from social media use, social media use^2 and the covariates of log household income, neighbourhood deprivation and year of data collection (*life satisfaction ratings ~ estimated social media use + estimated social media use^2 + log household income + neighbourhood deprivation + year of data collection)*. No statistical significance tests were carried out, and the statistical assumptions of the regression were not tested, further no correction for multiple comparisons were made.

It shows that the link between social media use and life satisfaction ratings is most negative in early adolescence compared with older adolescence and adulthood, but there are other ages when the relationship also becomes slightly more negative (e.g., males aged 26-29). Source data for this figure are provided as a Source Data file.

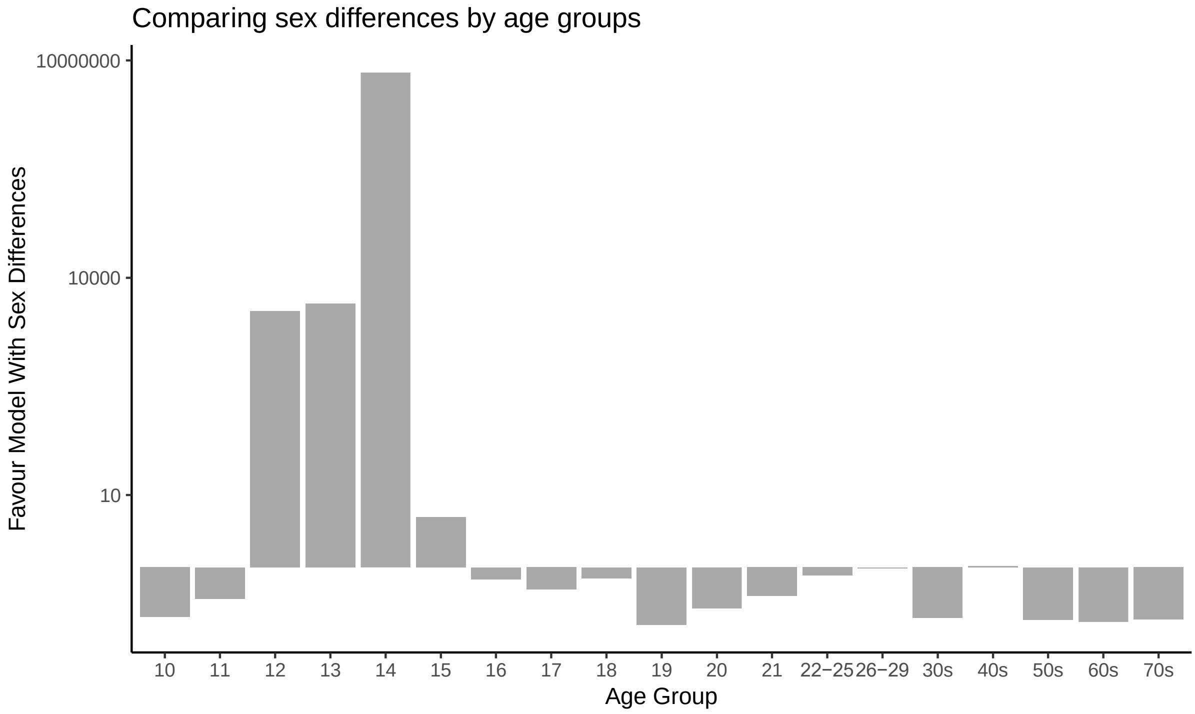

Supplementary Figure 3: Akaike weights ratios for different age groups, visualising at which age there is a preference for models that differentiate for sex when linking estimated social media use to life satisfaction ratings

Results from an Akaike weights procedure where two models were fit to data of different age groups. The models had the following form: *life satisfaction ~ a*social media + b*social media*^{2} (and including control variables for log household income, neighbourhood deprivation and year of data collection). One of the models allowed the parameters a and b to vary by sex, while the other model did not. The model fit indices (AIC) were compared using an Akaike weights procedure, and the ratio of the weights are plotted on the y-axis: the higher the bar, the more a model with sex differences is more likely to be a better model for the data than a model without sex differences at that age group. The graph gives no indication about which direction the sex difference is in. The y-axis is on a log scale to account for the rapidly increasing Akaike weights ratios. Source data for this figure are provided as a Source Data file.

Supplementary Methods 1

In the extension of the cross-sectional analyses, we analysed a range of questionnaires that were only completed by 10–15-year-olds in the Understanding Society survey and questionnaires completed by 13- and 14-year-olds in the Millennium Cohort Study.

Measures: Understanding Society

We constructed supplementary cross-sectional plots examining well-being and mental health questionnaires completed only by the younger adolescent sample (age 10-15 years). These included a longer well-being questionnaire at every wave which supplemented the single life satisfaction question used above with questions regarding satisfaction with school work, appearance, family, friends and school. Further, at every even wave adolescents completed an 8-item self-esteem scale and at every odd wave they filled in a 25-item Strengths and Difficulties Questionnaire (SDQ). The SDQ is a commonly used and widely validated measure for psychosocial functioning applied in school, home and clinical environments[1]. It encompasses five questions each about emotional symptoms, conduct problems, hyperactivity/inattention, peer relationship problems and prosocial behaviour (0 = not true, 1 = somewhat true, 2 = certainly true). We analysed conduct scores as a measure of externalising symptoms and emotional scores as an internalising symptoms measure.

Measures: Millennium Cohort Study

In the Millennium Cohort Study the SDQ measure was the same as in Understanding Society, but it was completed by the adolescent's caregiver. Furthermore the Millennium Cohort Study included a slightly different self-esteem measure: the 5-item Shortened Rosenberg Self-esteem scale[2]. Lastly the adolescents were asked to fill out a depressive symptoms scale in form of the "Mood and Feelings Questionnaire short form"[3], asking adolescents: "For each question please select the answer which reflects how you have been feeling or acting in the past two weeks." Responses were "I felt miserable or unhappy," "I didn't enjoy anything at all," "I felt so tired I just sat around and did nothing," "I was very restless," "I felt I was no good any more," "I cried a lot," "I found it hard to think properly or concentrate," "I hated myself," "I was a bad person," "I felt lonely," "I thought nobody really loved me," "I thought I could never be as good as other kids," and "I did everything wrong" (1 = not true, 2 = sometimes, 3 = true; scale subsequently reverse scored).

Latent Factors

As the questionnaires in the Understanding Society and Millennium Cohort Study surveys had more than one item, we first extracted latent factors and applied model comparison to examine measurement invariance across sex before plotting the latent factors' relation to estimated social media use. Due to the large sample size, chi-square tests for measurement invariance over sex routinely rejected the null hypothesis. We therefore claimed partial measurement invariance even if the null hypothesis was rejected, but when the completely freed model was preferred over the constrained model by the BIC (or when the BIC's are equal), which generally penalizes complexity more harshly than a likelihood ratio test.

We first analysed Understanding Society. To achieve partial measurement invariance for the well-being questionnaire, we allowed one item (satisfaction with school work) to vary across

sex as it loaded more highly for males than females: $\chi^2(4) = 15.4$, $p = 0.004$, BIC_{free} = 435,529, $BIC_{constrained}$ = 435,516; final model fit: $\chi^2(22)$ =1232.64, $p < 0.001$, RMSEA = 0.07, [0.06, 0.07], CFI = 0.95, SRMR = 0.04. We extracted two latent factors for SDQ, emotional/internalising symptoms and conduct/externalising symptoms respectively, and found that we needed to free "often accused of lying or cheating" (males > females) to achieve partial measurement invariance: $\chi^2(7) = 49.3$, $p < 0.001$, BIC_{free} = 229,089, $BIC_{constrained}$ = 229,089; final model fit: $\chi^2(75) = 1378.89$, $p < 0.001$, RMSEA = 0.05, [0.05, 0.05], CFI = 0.92, SRMR = 0.04. The self-esteem measure was treated as measurement invariant without having to free any additional item: $\chi^2(7) = 46.4$, $p < 0.001$, BIC_{free} = 154,119, $BIC_{constrained}$ = 154,115; final model fit: $\chi^2(47) = 2437.04$, $p < 0.001$, RMSEA = 0.10, [0.10, 0.10], CFI = 0.84, SRMR = 0.07.

For the Millennium Cohort Study data we extracted a latent factor of the well-being questionnaire, and freed satisfaction with friends and family to achieve partial measurement invariance as they loaded more highly for females than males: $\chi^2(3) = 19.9$, $p < 0.001$, BIC_{free} = 209,227, $BIC_{constrained}$ = 209,223; final model fit: $\chi^2(21) = 986.83$, $p < 0.001$, RMSEA = 0.09, [0.09, 0.10], CFI = 0.94, SRMR = 0.04. We extracted estimates for two latent factors from the SDQ: internalising symptoms (emotional subscale) and externalising symptoms (conduct subscale) (see methods above). We could treat it as measurement invariant without freeing additional items: $\chi^2(8) = 23.6$, $p = 0.003$, BIC_{free} = 154,832, $BIC_{constrained}$ = 154,808; final model fit: $\chi^2(76) = 1278.61$, $p < 0.001$, RMSEA = 0.05, [0.05, 0.06], CFI = 0.91, SRMR = 0.05. To achieve partial measurement for self-esteem in the Millennium Cohort Study, we freed "I am able to do things as well as most other people", "I am a person of value" and "I feel I have a number of good qualities" as they all load more highly for females than males: $\chi^2(1) = 0.27$, $p = 0.60$, BIC_{free} = 79,599, $BIC_{constrained}$ = 79,590; final model fit: $\chi^2(11)$ = 572.92, $p < 0.001$, RMSEA = 0.10, [0.09, 0.10], CFI = 0.97, SRMR = 0.03. For depressive symptoms measured using the Short Moods and Feelings Scale, we freed four out of the 13 items (all loadings: male > female): $\chi^2(8) = 48.3$, $p < 0.001$, BIC_{free} = 186,496, $BIC_{constrained}$ = 186,491; final model fit: $\chi^2(138) = 3995.81$, $p < 0.001$, RMSEA = 0.07, [0.07, 0.07], CFI = 0.92, SRMR = 0.05.

After extracting the latent factors for both Understanding Society and the Millennium Cohort Study, we plotted how they relate to estimated social media use by age (Supplementary Figure 4, Panel A). We also plotted each life satisfaction question's raw scores by social media use and age in Supplementary Figure 4, Panel B to examine whether a specific aspect of life satisfaction was more negatively related to estimated social media use (see also Figure 2).

Supplementary Methods 2

The BayesFactor package was used to calculate the Bayesian regression: "The vector of observations y is assumed to be distributed as: y ~ Normal(α 1 + Xβ, σ^2 I).

The joint prior on α,σ^2 is proportional to 1/σ^2, the prior on β is: β ~ Normal(0, N g σ^2(X'X)^{-1}), where g ~ InverseGamma(1/2,r/2)." [4]

Supplementary Results 1

We supplemented the analyses in the main manuscript by extracting factor scores for a wider set of well-being and mental health measures completed by 10–15-year-olds in our original dataset, Understanding Society, and 13–14-year-olds drawn from a different cohort (the UK Millennium Cohort Study, 11,724 participants). The limited age range did not allow us to compare these adolescents with other age groups, however the datasets present a similar pattern of sex differences across a wider range of psychosocial outcomes (Supplementary Figure 4, Panel A). In Understanding Society data, self-reported well-being showed the largest mean sex differences across ages 10-15 when compared to questionnaires such as self-esteem or depressive symptoms (Akaike weight of model with sex difference compared to model without sex difference: well-being 72.9%, self-esteem 55.3%, internalising symptoms 57.9%, externalising symptoms 56.2%). In the Millennium Cohort Study, the models differentiating between sex for well-being, depression and self-esteem were much more likely to be the better models of the data than those that did not differentiate for sex (100%), while those for externalising symptoms (21.3%) and internalising symptoms (35.5%) were not. For analyses of the correlations in Understanding Society over time see Supplementary Figure 5.

The well-being questionnaire (a questionnaire whose constituent questions include the satisfaction with life question analysed in the main manuscript, Figure 2) therefore seems to show the most substantial sex differences. Examining the constituent sub-questions that make up the wellbeing questionnaire (satisfaction with appearance, family, friends, school, school work and life), we found no evidence that a specific sub-component of life satisfaction was the lone driver of these sex differences (Supplementary Figure 4, Panel B; Figure 2). See the main manuscript and Supplementary Figure 6 for further analyses.

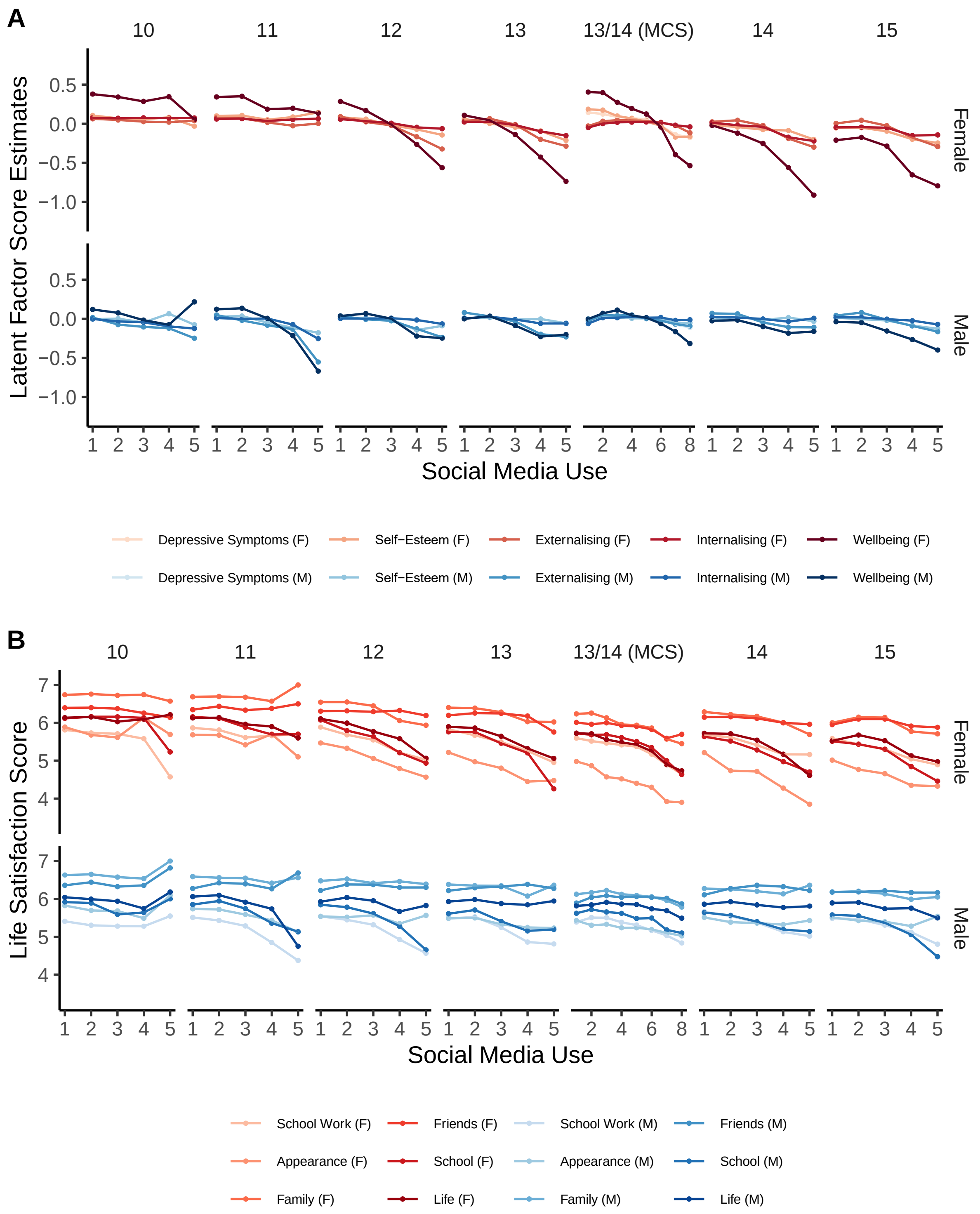

Supplementary Figure 4: The cross-section relationship between social media use, well-being and mental health (ages 10-15)

The Figure shows the cross-sectional relation between estimated social media use and well-being & mental health measures at ages 10–15 (Understanding Society dataset, US; 10,019 participants and 24,698 measurement occasions) and ages 13–14 (Millennium Cohort Study dataset, MCS; 11,724 participants). Panel A: Cross-sectional correlation between estimated social media use and latent factors of a variety of well-being or mental health measures at

ages 10-15 for US and 13-14 for MCS. Depressive Symptoms were only measured in the MCS dataset. The relationships for males and females are presented separately: females = top/red, males = bottom/blue. The 95% confidence intervals represent the lower and upper Gaussian confidence limits around the mean based on the t-distribution. Panel B: Cross-sectional correlation between estimated social media use and raw scores of six sub-components of life satisfaction (which make up the life satisfaction scale analysed in Panel A) at ages 10-15 for US and 13-14 for MCS. The relationships are also presented separately for males and females. The 95% confidence intervals represent the lower and upper Gaussian confidence limits around the mean based on the t-distribution.

The figure shows clear sex differences present in both US and MCS datasets, with life satisfaction ratings showing a more negative relation to estimated social media use in adolescent females compared with adolescent males. The other mental health and well-being measurements show less prominent differences. Further, there is no one sub-component of life satisfaction that predominately drives the sex differences found. Source data for this figure are provided as a Source Data file.

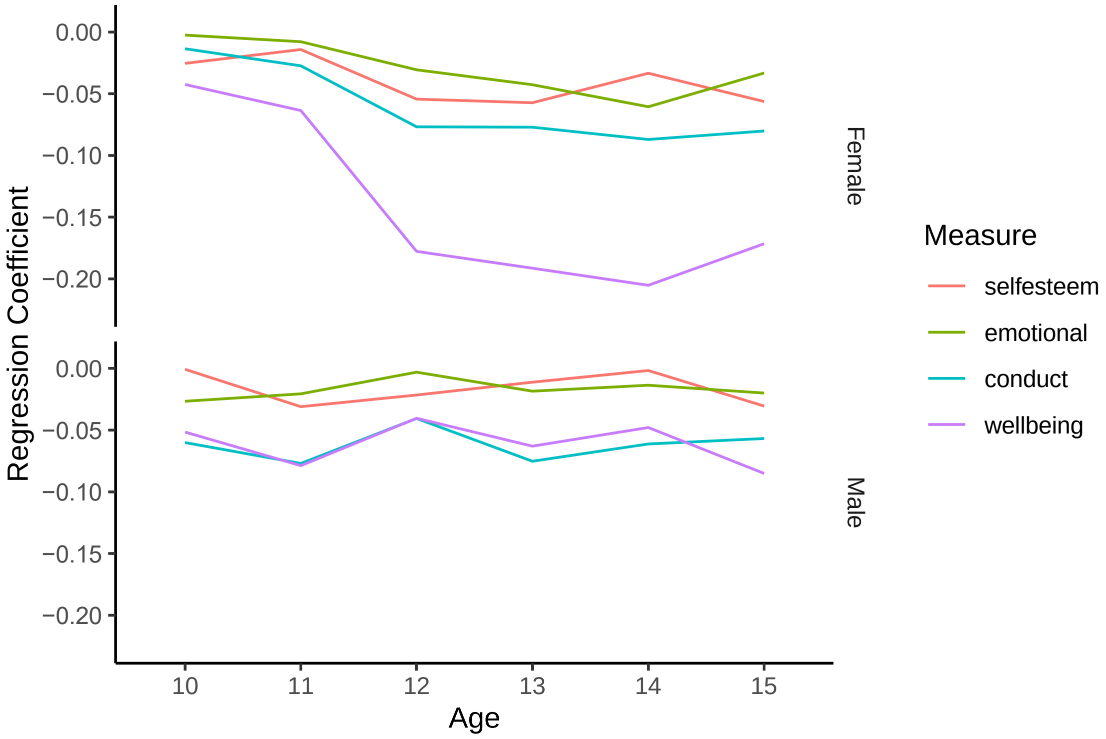

Supplementary Figure 5: Plot of the cross-sectional correlation between estimated social media use and different mental health and well-being measures (estimated using latent factors) by age and sex

The plot shows that the cross-sectional correlation between estimated social media use and mental health and well-being measures stays relatively stable across early adolescence, except wellbeing whose correlation becomes more negative in females during the period. Using the confidence intervals that represent standard errors around the mean, one can interpret which measures' relation to social media use is different statistically. For example, across both sexes and most ages, well-being and conduct symptoms are more negatively related to social media use than other measures. No correction for multiple comparisons was made. Source data for this figure are provided as a Source Data file.

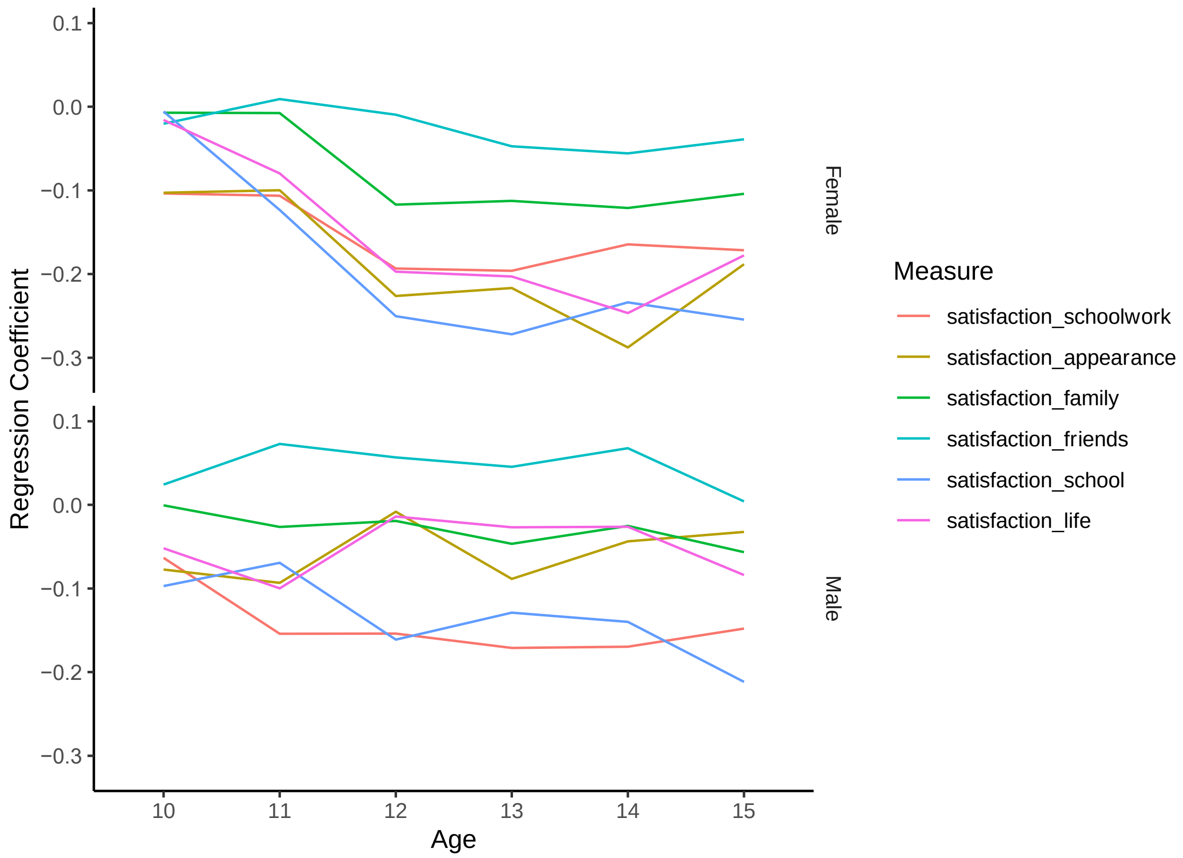

Supplementary Figure 6: Plot of the cross-sectional correlation between estimated social media use and different life satisfaction measures (raw scores) by age and sex

The plot shows that there are differences in how estimated social media use relates to life satisfaction measures across age and sex. Using the confidence intervals that represent standard errors around the mean, one can interpret which measures' relationship with social media use is different statistically. For example, in females the relationship becomes more negative for satisfaction with appearance, life, school and school work; while the relationship between estimated social media use and satisfaction with family and friends is less negative than other measures and stays relatively stable over age. No correction for multiple comparisons was made. Source data for this figure are provided as a Source Data file.

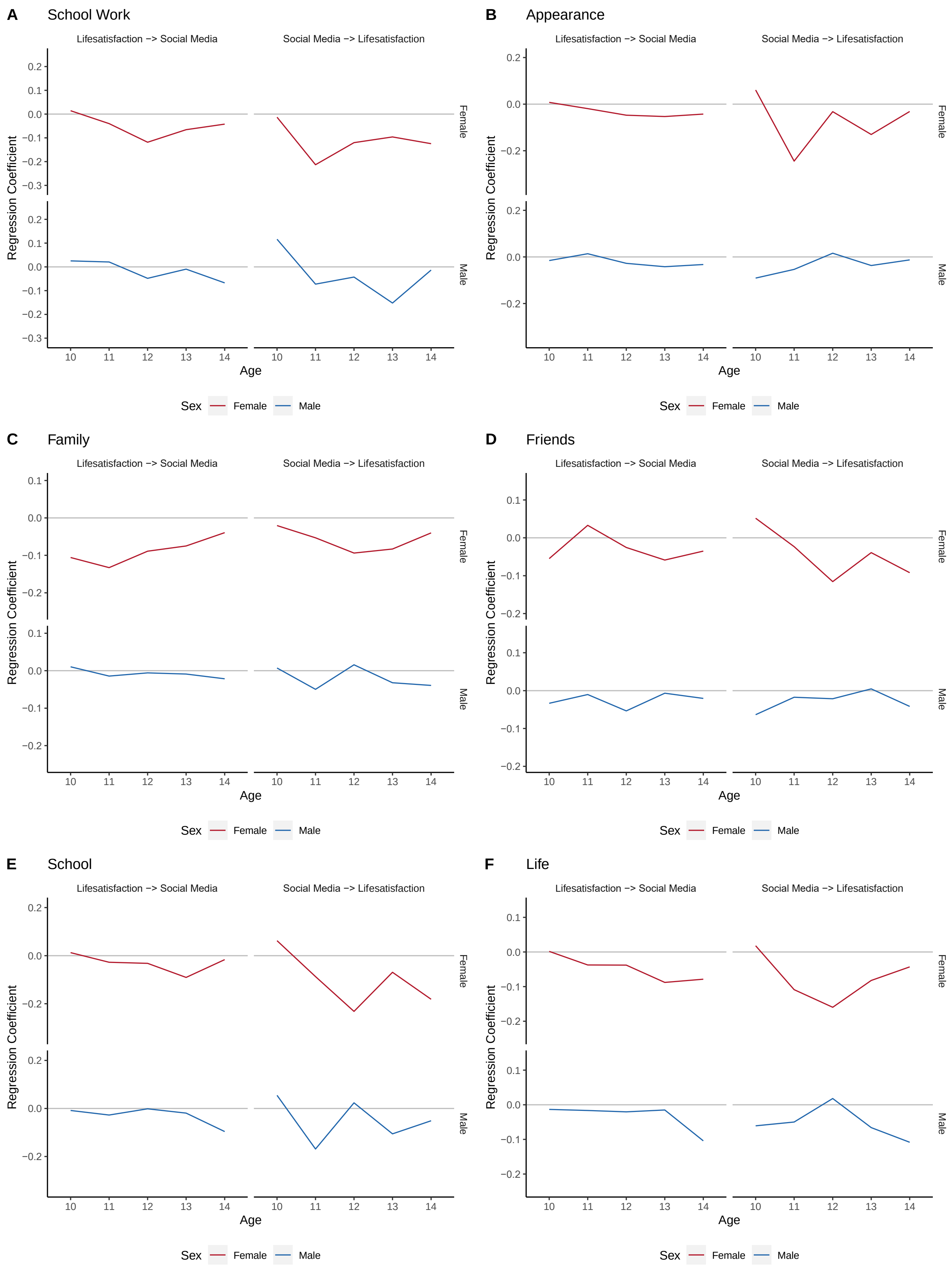

Supplementary Figure 7: Results from Random Intercept Cross-Lagged Panel Models (RI-CLPM) of estimated social media use and different components of life satisfaction for

participants of the Understanding Society dataset aged 10-15. Results from both cross-lagged paths of a RI-CLPM where those paths were free to vary across age/sex. The different panels represent different components of satisfaction with life: a = satisfaction with school work, b = satisfaction with appearance, c = satisfaction with family, d = satisfaction with friends, e = satisfaction with school and f = satisfaction with life. Results are unstandardised and split by path (left: deviations from expected life satisfaction ratings at that age predicting deviations from expected social media use one year later; right: deviations from expected social media use at that age predicting deviations from expected life satisfaction ratings one year later) and sex (female = top/red, male = bottom/blue). The ribbon represents the 95% Confidence Interval around the point estimate. No correction for multiple comparisons was made. Source data for this figure are provided as a Source Data file.

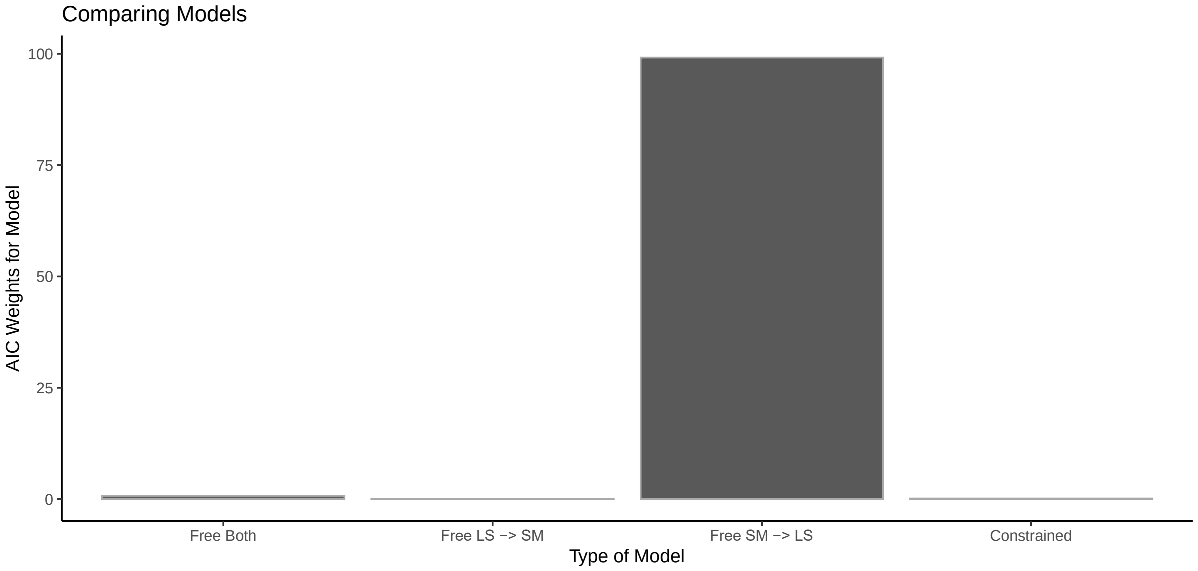

Supplementary Figure 8: Akaike weights showing which Random-Intercept Cross Lagged Panel Model is preferred for modelling estimated social media use and life satisfaction ratings in the Understanding Society dataset (ages 10-21).

Results from an Akaike weights procedure where four different Random-Intercept Cross Lagged Panel Models were fitted to the data: 1) a model that allowed both cross lagged paths to vary by sex and age ("Free Both"); 2) a model that allowed only the cross lagged path from life satisfaction ratings (LS) to estimated social media (SM) use to vary by sex and age ("Free LS -> SM"); 3) a model that allowed only the cross lagged path from estimated social media use to life satisfaction ratings to vary by sex and age ("Free SM -> LS"); and 4) a model that allowed none of the cross lagged paths to vary by sex and age ("Constrained"). The higher the bar the more the model is preferred over the others. Source data for this figure are provided as a Source Data file.

	Number of Measurement Occasions
Age	
10	3895
11	4048
12	4149
13	4229
14	4272
15	4105
16	4740
17	4889
18	4804
19	4641
20	4435
21	4349
22	4213
23	4052
24	3897
25	3806
26	3742
27	3789
28	3828
29	3849
30	4055
31	4205
32	4362
33	4434

34	4546
35	4630
36	4652
37	4712
38	4835
39	4867
40	5042
41	5102
42	5325
43	5459
44	5563
45	5568
46	5622
47	5558
48	5510
49	5461
50	5399
51	5339
52	5281
53	5139
54	5111
55	4997
56	4842
57	4715
58	4628
59	4477
60	4454

61	4380
62	4309
63	4370
64	4476
65	4443
66	4398
67	4298
68	4263
69	4142
70	3873
71	3577
72	3345
73	3142
74	2979
75	2837
76	2632
77	2450
78	2269
79	2105
Sex	
Male	142010
Female	163900

Supplementary Table 1: Number of measurement occasions by age and sex in Understanding Society data analysed cross-sectionally (ages 10-79). The number of male and female measurement occasions per age group can be found on the OSF repository (1d_descriptives_us.html)

	Number of Measurement Occasions	Number of Measurement Occasions [Female]	Number of Measurement Occasions [Male]
Age			
10	3895	1953	1942
11	4048	2014	2034
12	4149	2042	2107
13	4229	2118	2111
14	4272	2106	2166
15	4105	2072	2033
16	4740	2398	2342
17	4889	2486	2403
18	4804	2456	2348
19	4641	2393	2248
20	4435	2289	2146
21	4349	2274	2075
Sex			
Male	25955		
Female	26601		

Supplementary Table 2: Number of measurement occasions by age and sex in younger and older adolescents in Understanding Society data analysed longitudinally (ages 10-21).

	Number of Measurement Occasions	Number of Measurement Occasions [Female]	Number of Measurement Occasions]
Age			
13	2864	1416	1448
14	8860	4437	4423
Sex			
Male	5871		
Female	5853		

Supplementary Table 3: Number of measurement occasions by age and sex in the Millennium Cohort Study

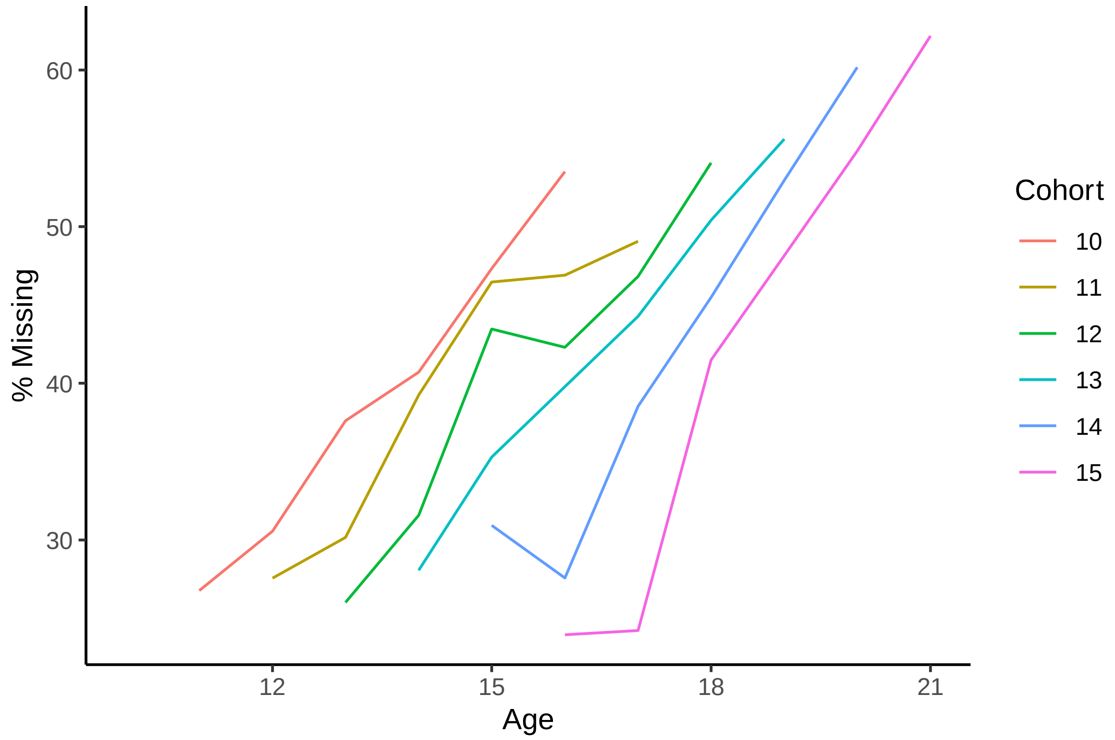

Supplementary Figure 9: Missing Data (%) by Age

To examine how missingness develops over both younger and older adolescent cohorts in Understanding Society we investigated the percentage missingness in those age cohorts who remained in the adolescent survey for all seven waves of data collection (those aged 10, 11, 12, 13, 14 and 15 during the first wave). We find that missingness increases over time, but that it increases faster in later age cohorts. This shows that attrition is greater at older ages. Source data for this figure are provided as a Source Data file.

	Age 10			Age 11			Age 12			Age 13			Age 14			Age 15		
Predictors	OR	CI	*p*	OR	CI	*p*	OR	CI	*p*	OR	CI	*p*	OR	CI	*p*	OR	CI	*p*
Intercept	1.94	0.12-32.21	0.641	0.03	0.00-0.52	0.018	0.03	0.00-0.32	0.005	0.18	0.01-2.02	0.171	0.02	0.00-0.20	0.002	2.12	0.21-20.98	0.520
Life satisfaction	0.86	0.75-0.98	0.023	1.04	0.91-1.20	0.570	1.09	0.96-1.25	0.182	0.96	0.84-1.10	0.582	1.05	0.92-1.20	0.488	0.99	0.86-1.15	0.904
Social media	0.81	0.64-1.01	0.064	1.04	0.86-1.26	0.673	0.86	0.73-1.02	0.089	0.91	0.77-1.07	0.253	0.84	0.72-0.98	0.028	0.85	0.73-1.00	0.051
Deprivation (IMD)	1.05	0.99-1.11	0.077	1.01	0.96-1.07	0.637	1.00	0.94-1.05	0.854	1.01	0.95-1.07	0.751	1.04	0.99-1.10	0.138	1.03	0.97-1.09	0.310
Household income	1.04	0.74-1.46	0.831	1.55	1.08-2.26	0.019	1.55	1.14-2.14	0.006	1.29	0.96-1.76	0.095	1.60	1.17-2.21	0.003	0.92	0.70-1.21	0.536
Sex (male)	0.74	0.54-1.00	0.047	0.67	0.49-0.91	0.010	0.62	0.46-0.83	0.001	0.71	0.52-0.96	0.026	0.78	0.58-1.05	0.107	0.57	0.42-0.78	<0.001
Observations	700			681			764			727			768			724		
R^2 Tjur	0.022			0.020			0.028			0.013			0.032			0.022		

Supplementary Table 4: Examining selective attrition using ordinal regression predicting whether a participant present in wave 1 had not dropped out the survey at wave 7 from a variety of predictors.

In addition to the analyses in Supplementary Figure 9, we examined selective attrition further in those age cohorts who remained in the Understanding Society adolescent survey for all seven waves of data collection (those aged 10, 11, 12, 13, 14 and 15 during the first wave). To do so, we coded those adolescents as '1' who had data for wave 1 and wave 7, and those as '0' for those who had data for only wave 1 and not wave 7. We then used ordinal regression to regress this attrition variable onto the predictor variables of sex, mean income (inc), mean index of multiple deprivation (imd), social media use at wave 1 (sm) and life satisfaction at wave 1 (ls). The test was two-tailed (OR = odds ratios) We found that the only consistent predictor of attrition is sex (males showing higher levels of attrition). Further, for half of the age cohorts lower income predicts higher levels of attrition. No correction for multiple comparisons was made.

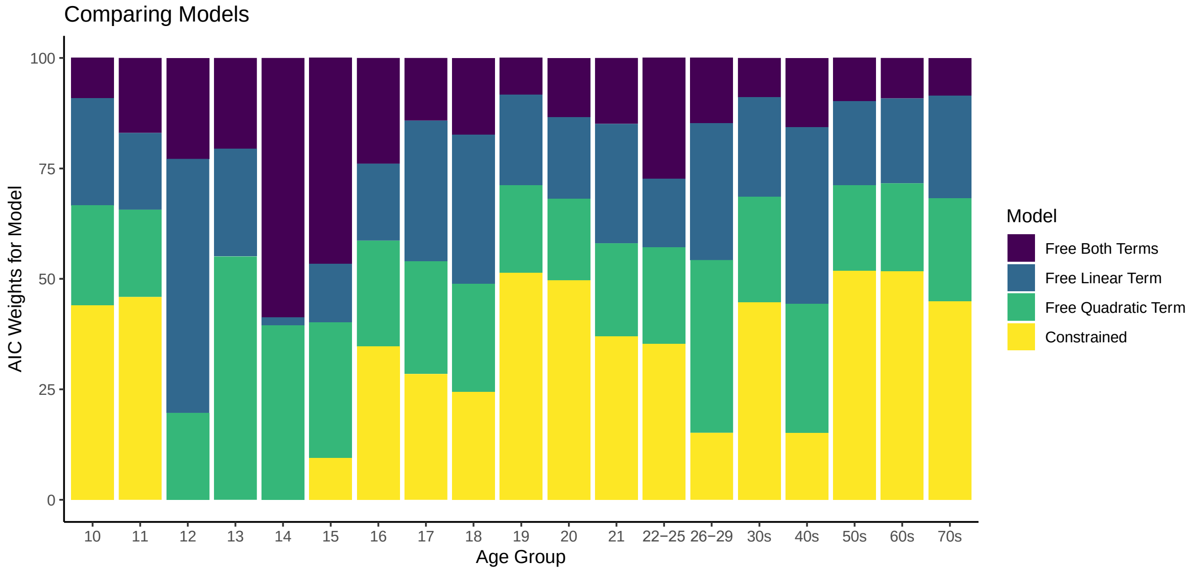

Supplementary Figure 10: Akaike weights showing at which age groups there is a preference for models linking social media use and life satisfaction that differentiate for sex in either linear, quadratic or both terms.

This graph extends the results shown in Supplementary Figure 1 and 3. In particular, it shows the results from an Akaike weights procedure where the same model was fit to the data*: life satisfaction ~ a*social media + b*social media*^{2}, and further control variables of household income, neighbourhood deprivation and year of data collection. However, the parameters a and b were either both allowed to vary by sex ("Free Both Terms"), only one was allowed to vary by sex ("Free Linear Term"/"Free Quadratic Term") or none was allowed to vary by sex ("Constrained"). The y-axis shows the Akaike weights for each of the four models. Source data for this figure are provided as a Source Data file.

Supplementary References

1. Goodman, R., Ford, T., Simmons, H., Gatward, R. & Meltzer, H. Using the Strengths and Difficulties Questionnaire (SDQ) to screen for child psychiatric disorders in a community sample. *The British Journal of Psychiatry* **177**, 534–539 (2000).
2. Robins, Richard. W., Hendin, Holly. M. & Trzesniewski, Kali. H. Measuring Global Self-Esteem: Construct Validation of a Single-Item Measure and the Rosenburg Self-Esteem Scale. *Personality and Social Psychology Bulletin* **27**, 151–161 (2001).
3. Thapar, A. & McGuffin, P. Validity of the shortened Mood and Feelings Questionnaire in a community sample of children and adolescents: a preliminary research note. Psychiatry Research 81, 259–268 (1998)
4. Morey, R.D. & Rouder, J.N. BayesFactor: Computation of Bayes Factors for common designs. R package v0.9.12-2. (2015)
