# Supplementary material for: Windows of developmental sensitivity to social media
Source: Nat Commun. 2022 Mar 28;13:1649. doi: 10.1038/s41467-022-29296-3 (PMC8960761; doi:10.1038/s41467-022-29296-3)
Supplement: Supplementary file 3 — Reporting Summary [file 41467_2022_29296_MOESM3_ESM.pdf]

nature research

Corresponding author(s): Amy Orben

Last updated by author(s): 2022/01/12

Reporting Summary

Nature Research wishes to improve the reproducibility of the work that we publish. This form provides structure for consistency and transparency in reporting. For further information on Nature Research policies, see our Editorial Policies and the Editorial Policy Checklist.

Statistics

For all statistical analyses, confirm that the following items are present in the figure legend, table legend, main text, or Methods section.

n/a	Confirmed	
☐	☒	The exact sample size (*n*) for each experimental group/condition, given as a discrete number and unit of measurement
☐	☒	A statement on whether measurements were taken from distinct samples or whether the same sample was measured repeatedly
☐	☒	The statistical test(s) used AND whether they are one- or two-sided<br>*Only common tests should be described solely by name; describe more complex techniques in the Methods section.*
☐	☒	A description of all covariates tested
☐	☒	A description of any assumptions or corrections, such as tests of normality and adjustment for multiple comparisons
☐	☒	A full description of the statistical parameters including central tendency (e.g. means) or other basic estimates (e.g. regression coefficient) AND variation (e.g. standard deviation) or associated estimates of uncertainty (e.g. confidence intervals)
☐	☒	For null hypothesis testing, the test statistic (e.g. *F*, *t*, *r*) with confidence intervals, effect sizes, degrees of freedom and *P* value noted<br>*Give P values as exact values whenever suitable.*
☐	☒	For Bayesian analysis, information on the choice of priors and Markov chain Monte Carlo settings
☒	☐	For hierarchical and complex designs, identification of the appropriate level for tests and full reporting of outcomes
☐	☒	Estimates of effect sizes (e.g. Cohen's *d*, Pearson's *r*), indicating how they were calculated

Our web collection on statistics for biologists contains articles on many of the points above.

Software and code

Policy information about availability of computer code

Data collection	The authors did not collect the data.
Data analysis	The code for all analysis and data cleaning are available on the Open Science Framework: https://osf.io/fzspx/?view_only=10a35250aadd4e17a7659cb0efa30e50. A list of r packages and version numbers, as well as further session information, are available at the end of each of our scripts on the OSF, this includes the r package lavaan (0.6-7.1571) we used for our longitudinal modelling.

For manuscripts utilizing custom algorithms or software that are central to the research but not yet described in published literature, software must be made available to editors and reviewers. We strongly encourage code deposition in a community repository (e.g. GitHub). See the Nature Research guidelines for submitting code & software for further information.

Data

Policy information about availability of data

All manuscripts must include a data availability statement. This statement should provide the following information, where applicable:

- Accession codes, unique identifiers, or web links for publicly available datasets
- A list of figures that have associated raw data
- A description of any restrictions on data availability

The Understanding Society and Millennium Cohort Study data used in this study have been deposited in the UK Data Service database. They are accessible after registration with the UK Data Service and completion of an End User Agreement (Understanding Society: University of Essex, Institute for Social and Economic Research, NatCen Social Research, Kantar Public. (2019). Understanding Society: Waves 1-9, 2009-2018 and Harmonised BHPS: Waves 1-18, 1991-2009. [data collection]. 12th Edition. UK Data Service. SN: 6614, http://doi.org/10.5255/UKDA-SN-6614-13; Millennium Cohort Study: University of London, Institute of Education, Centre for Longitudinal Studies. (2020). Millennium Cohort Study: Sixth Survey, 2015. [data collection]. 6th Edition. UK Data Service. SN: 8156, http://doi.org/10.5255/UKDA-SN-8156-6.). Some of the data used in our study might not be accessible to users outside the UK. Please check the terms and conditions for

each of the datasets prior to use on the UK Data Service. The Special License data necessary to calculate Index of Multiple Deprivation in Understanding Society are available after an approval procedure through the UK Data Service. The figure and table data generated in this study are provided in the Source Data file. Amy Orben accessed all datasets.

Field-specific reporting

Please select the one below that is the best fit for your research. If you are not sure, read the appropriate sections before making your selection.

☐ Life sciences ☒ Behavioural & social sciences ☐ Ecological, evolutionary & environmental sciences

For a reference copy of the document with all sections, see nature.com/documents/nr-reporting-summary-flat.pdf

Behavioural & social sciences study design

All studies must disclose on these points even when the disclosure is negative.

Study description

Understanding Society: Quantative panel study

Millennium Cohort Study: Quantitative Cohort Study

Research sample

Understanding Society: a longitudinal study following approximately 40,000 British households. The study sample was recruited to ensure accurate representation of the UK population. Started in 2009, its annual waves of data collection each span two years; we used 7 waves of data from between 2011 and 2018 released in February 2020 (the two first waves were excluded as parts of the sample were not asked to completed social media related questions). All household members between 10 and 15 years filled out a youth survey, while those 16 and over filled out an adult survey. 16-21 year-olds further completed a short young adult supplement with additional questions.

Millennium Cohort study: a birth cohort study of a sample of around 11,000 young people born between September 2000 and January 20013. In this study we only used the most recent wave of the data collected in 2015, when the majority of respondents were 13 or 14 years old. This made the two datasets comparable (e.g. in terms of the prevalence and use of social media) to subsections of our Understanding Society sample.

Study sample demographic information can be found in Supplementary Tables 1, 2 and 3.

Sampling strategy

Understanding Society: the sample design consists of multiple sample components (e.g., the largest population is the General Population Sample which is clustered and stratified). The sampling strategy is defined in detail in Lynn (2009): Peter Lynn (2009) Sample design for Understanding Society, Understanding Society Working Paper 2009-01, Colchester: University of Essex (https://www.understandingsociety.ac.uk/sites/default/files/downloads/working-papers/2009-01.pdf). Further information about the sample design can be found under the following link: https://www.understandingsociety.ac.uk/sites/default/files/downloads/documentation/user-guides/mainstage/sample-design.pdf

Millennium Cohort Study: MCS cohort members were recruited from the UK population born over a 17 month period in September 2000 to January 2002, using a sampling strategy where areas of residence were selected and all children born in the eligible period were recruited. The sampling strategy is defined in detail in Plewis (2007): Ian Plewis (2007) The Millennium Cohort Study: Technical Report on Sampling (4th Edition), London: Centre for Longitudinal Studies, Bedford Group for Lifecourse and Statistical Studies, Institute of Education, University of London (https://cls.ucl.ac.uk/wp-content/uploads/2017/07/Technical-Report-on-Sampling-4th-Edition-August-2007.pdf).

Data collection

Understanding Society: interviews were most often carried out face-to-face in people's homes by trained interviewers or respondents completed the survey online. A detailed overview of fieldwork procedures can be found using the following link: https://www.understandingsociety.ac.uk/documentation/mainstage/user-guides/main-survey-user-guide/fieldwork-procedures-2009. Furthermore, fieldwork documents can be found here: https://www.understandingsociety.ac.uk/documentation/mainstage/fieldwork-documents.

Millennium Cohort Study: Extensive information about study data collection and design can be found in Joshi, H. and Fitzsimons, E. (2016) The Millennium Cohort Study: the making of a multi-purpose resource for social science and policy. Longitudinal and Life Course Studies, 7(4), 409-430. (http://dx.doi.org/10.14301/llcs.v7i4.410). Further there are two sourcebooks available on the topic: Children of the 21st century: from birth to nine months and Children of the 21st century: the first 5 years (edited respectively by Dex and Joshi, 2005, and Hansen, Joshi, and Dex, 2010).

Timing

Understanding Society: Each wave of data collection was 24 months long, however there was follow up for interviews that could not be conducted during this period. The waves analysed in this study were collected during the following times: 1 January 2011 - 30 June 2013; 1 January 2012 - 30 June 2014; 1 January 2013 - 30 June 2015; 1 January 2014 - 30 June 2016; 1 January 2015 - 30 June 2017; 1 January 2016 - 30 June 2018; 1 January 2017 - 30 June 2019. More details about the survey timeline can be found on the following two links: https://www.understandingsociety.ac.uk/documentation/mainstage/user-guides/main-survey-user-guide/survey-timeline and https://www.understandingsociety.ac.uk/documentation/mainstage/survey-timeline

Millennium Cohort Study: The wave of the Millennium Cohort Study analysed in this paper was conducted between the 15 January 2015 and 30 March 2016. More detail about the fieldwork timings can be found in Emla Fitzsimons (2017) Millennium Cohort Study Sixth Survey 2015-2016 User Guide (First Edition), London: Institute of Education, University College London.

Data exclusions

Understanding Society: we excluded a variety of participants from the dataset. Firstly, we excluded people aged over 80, because

Data exclusions	social media use was very low at higher ages (excluding 14,357 measurement occasions). Next, we excluded those who filled out the incorrect survey, i.e. the under 10 year olds or over 15 year olds who filled out the youth survey, or the under 16 year olds who filled out the adult survey (excluding 47 and 45 measurement occasions, respectively). We further excluded those participants who completed a questionnaire twice in one age category (we only excluded the second time the questionnaire was filled out in one age category, excluding 9,253 measurement occasions) and those whose gender was NA (25 measurement occasions; there were too few datapoints to treat this as a separate category). The latter could be due to them switching genders in the data collection frame, or refusing to answer the question. These exclusions left a sample of 72,281 participants over 7 waves (1 wave = 12,441 participants, 2 waves = 8,778 participants, 3 waves = 8,797 participants, 4 waves = 7,425 participants, 5 waves = 7,096 participants, 6 waves = 9,895 participants, 7 waves = 17,849 participants). The adolescent (10-21 years) sample used for longitudinal modelling consisted of 17,403 participants and 52,527 measurement occasions.<br><br>Millennium Cohort Study: In the Millennium Cohort Study we excluded those not aged 13 or 14 (160 participants), leaving 11,724 participants.<br><br>We did not apply sampling weights in either dataset and therefore cannot fully generalise our results to the whole UK population.
Non-participation	Understanding Society: individual response rates for all samples in Understanding Society can be found using the following link: https://www.understandingsociety.ac.uk/sites/default/files/downloads/documentation/user-guides/mainstage/responsetables.pdf The dropout has the potential to impact our results.<br><br>Millennium Cohort Study: 19,243 families were potentially eligible for MCS6, 3,828 families were "not issued into the field (due to death or emigration; permanent refusal; untraceability; sensitive situations)". 11,726 families were collected successfully (60.9%). More detail about non-participation can be found in Emla Fitzsimons (2017) Millennium Cohort Study Sixth Survey 2015-2016 User Guide (First Edition), London: Institute of Education, University College London.
Randomization	The study was not experimental and participants were not allocated into groups, therefore randomisation is not relevant for this study.

Reporting for specific materials, systems and methods

We require information from authors about some types of materials, experimental systems and methods used in many studies. Here, indicate whether each material, system or method listed is relevant to your study. If you are not sure if a list item applies to your research, read the appropriate section before selecting a response.

Materials & experimental systems

n/a	Involved in the study	
☒	☐	Antibodies
☒	☐	Eukaryotic cell lines
☒	☐	Palaeontology and archaeology
☒	☐	Animals and other organisms
☐	☒	Human research participants
☒	☐	Clinical data
☒	☐	Dual use research of concern

Methods

n/a	Involved in the study	
☒	☐	ChIP-seq
☒	☐	Flow cytometry
☒	☐	MRI-based neuroimaging

Human research participants

Policy information about studies involving human research participants

Population characteristics	See above
Recruitment	Understanding Society: the sample design consists of multiple sample components each recruited in different ways (e.g., the largest population is the General Population Sample which is clustered and stratified). The sampling strategy is defined in detail in Lynn (2009): Peter Lynn (2009) Sample design for Understanding Society, Understanding Society Working Paper 2009-01, Colchester: University of Essex (https://www.understandingsociety.ac.uk/sites/default/files/downloads/working-papers/2009-01.pdf). Further information about the sample design can be found under the following link: https://www.understandingsociety.ac.uk/sites/default/files/downloads/documentation/user-guides/mainstage/sample-design.pdf<br><br>Millennium Cohort Study: MCS cohort members were recruited from the UK population born over a 17 month period in September 2000 to January 2002, using a sampling strategy where areas of residence were selected and all children born in the eligible period were recruited. The sampling strategy is defined in detail in Plewis (2007): Ian Plewis (2007) The Millennium Cohort Study: Technical Report on Sampling (4th Edition), London: Centre for Longitudinal Studies, Bedford Group for Lifecourse and Statistical Studies, Institute of Education, University of London (https://cls.ucl.ac.uk/wp-content/uploads/2017/07/Technical-Report-on-Sampling-4th-Edition-August-2007.pdf).
Ethics oversight	The University of Essex Ethics Committee has approved all data collection on the Understanding Society main study and innovation panel waves, including asking consent for all data linkages except to health records. Ethical approval for the Millennium Cohort Study was given by the UK National Health Service (NHS) London, Northern, Yorkshire and South-West Research Ethics Committees (MREC/01/6/19, MREC/03/2/022, 05/MRE02/46, 07/MRE03/32). No additional ethical approval was needed for this study.

Note that full information on the approval of the study protocol must also be provided in the manuscript.
